# Thermally and field-driven mobility of emergent magnetic charges in square artificial spin ice

**DOI:** 10.1038/s41598-019-52460-7

**Published:** 2019-11-05

**Authors:** Sophie A. Morley, Jose Maria Porro, Aleš Hrabec, Mark C. Rosamond, Diego Alba Venero, Edmund H. Linfield, Gavin Burnell, Mi-Young Im, Peter Fischer, Sean Langridge, Christopher H. Marrows

**Affiliations:** 1 School of Physics and Astronomy, University of Leeds, Leeds, LS2 9JT United Kingdom; 2 Physics Department, University of California Santa Cruz, Santa Cruz, CA 95064 USA; 3 Advanced Light Source, Lawrence Berkeley National Laboratory, 1 Cyclotron Road, Berkeley, CA 94720 USA; 4 ISIS Neutron and Muon Source, STFC Rutherford Appleton Laboratory, Chilton, Didcot, Oxon. OX11 0QX United Kingdom; 5 BCMaterials, Basque Center for Materials, Applications and Nanostructures, 48940 Leioa, Spain; 6 Ikerbasque, Basque Foundation for Science, 48013 Bilbao, SpainIkerbasque, Basque Foundation for Science, 48013 Bilbao, Spain; 7 School of Electronic and Electrical Engineering, University of Leeds, Leeds, LS2 9JT United Kingdom; 8 Center for X-ray Optics, Lawrence Berkeley National Laboratory, 1 Cyclotron Road, Berkeley, CA 94720 USA; 9 Daegu Gyeongbuk Institute of Science and Technology, Daegu, 711-873 Korea; 10 Materials Sciences Division, Lawrence Berkeley National Laboratory, 1 Cyclotron Road, Berkeley, CA 94720 USA

**Keywords:** Imaging techniques, Magnetic properties and materials, Magnetic properties and materials

## Abstract

Designing and constructing model systems that embody the statistical mechanics of frustration is now possible using nanotechnology. We have arranged nanomagnets on a two-dimensional square lattice to form an artificial spin ice, and studied its fractional excitations, emergent magnetic monopoles, and how they respond to a driving field using X-ray magnetic microscopy. We observe a regime in which the monopole drift velocity is linear in field above a critical field for the onset of motion. The temperature dependence of the critical field can be described by introducing an interaction term into the Bean-Livingston model of field-assisted barrier hopping. By analogy with electrical charge drift motion, we define and measure a monopole mobility that is larger both for higher temperatures and stronger interactions between nanomagnets. The mobility in this linear regime is described by a creep model of zero-dimensional charges moving within a network of quasi-one-dimensional objects.

## Introduction

Artificial spin ices (ASI) are arrays of nanomagnetic islands that are effectively single domain and so have bistable Ising-like macrospin states. They have emerged as a playground to study the statistical mechanics of frustration phenomena in a simplified setting^1^. The particular beauty of these systems is that one is able to continuously tune various parameters such as interaction strength between nanomagnets^2^, material^3^, and array topology and geometry^4,5^, making them designer metamaterials. There has been great interest in the past in the attainment and preparation of low energy or ground states and the impact different parameters have on this, imaged as athermal snapshots of quenched thermally equilibrated states^2,6–8^. More recently, partly or fully thermalised systems undergoing fluctuation and relaxation^9–13^ and temperature driven phase transitions^14^ have been studied. Here we study the thermally-activated dynamics a square 2D ASI display in response to an external drive field through the direct imaging of the microstates involved.

**Figure 1:**
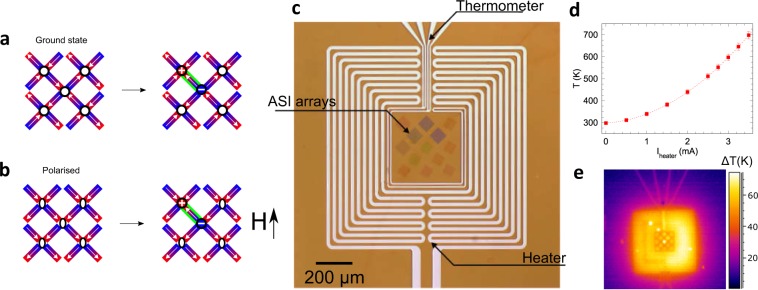
Monopole-antimonopole pair creation and on-membrane heater. Starting from a lattice of ice-rule obeying vertices (left) it is possible to flip a connecting macrospin between two vertices (outlined in green on the right) to create two Type 3 vertices, known as an emergent monopole-antimonopole pair. This can be done from (**a)** the Type 1 ground state vertices by thermal excitation or (**b)** from a pair of diagonally field-polarised Type 2 vertices by applied field. (**c)** An optical micrograph of the on-membrane heater and thermometer. The meandering Pt wires are counter-wound to avoid induced magnetic field at the sample and the ASI arrays were patterned in the centre of the design: here 13 separate arrays are visible with various lattice constants. (**d)** The temperature of the thermometer, obtained as a function of the heater current, *I*_heater_. (**e**) An infra-red thermal microscope image taken at *I*_heater_ = 2.3 mA (*T* = 467 K), showing an even temperature distribution across almost all the arrays on the membrane.

**Figure 2:**
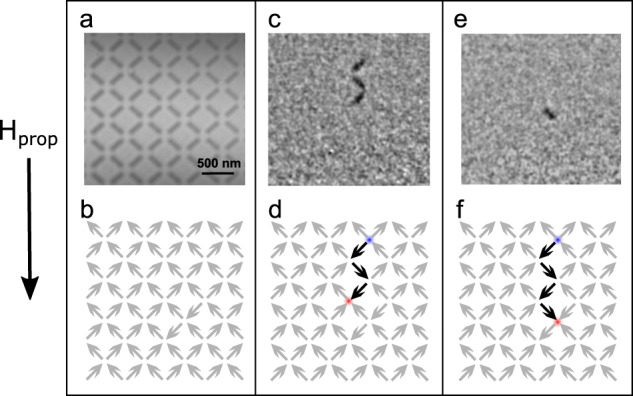
Imaging monopole motion. (**a)** The raw absorption image for the 400 nm lattice spacing sample and (**b)** a schematic of the initial, pure T_2_, diagonally field-polarised state. (**c)** A difference image where three islands have reversed and (**d)** the schematic of the corresponding injected monopole-antimonopole (T_3_) pair indicated with a red and blue circle at the ends of the string of reversed islands (T_1_ vertices) in black. (**e)** The next consecutive difference image where another island has reversed and (**f)** the cumulative motion of the monopole-antimonopole pair, schematically showing the creation of an additional T_1_ vertex and growth of the Nambu string.

In particular, we take advantage of the concept that excitations in the pyrochlore spin ice materials have been described as emergent magnetic monopole-like charges^15^. Experimentally confirmed in the pyrochlore systems^16,17^, emergent magnetic monopoles and dimensional reduction have been observed in kagome^18,19^ and square^20^ artificial spin ices at the coercive field during athermal reversal or purely thermal in a relaxation experiment^8,10^. Most recently, Debye-Huckel type behaviour of purely thermal emergent monopoles in ASI has been reported, confirming their Coulomb-like properties^21^. The picture for the square ices is the same as in the pyrochlores: these systems obey an ‘ice rule’, defined as the lowest energy arrangements consisting of two moments pointing into the fourfold vertex and two pointing out, leading to no net charge at the vertex in a dumbbell model. Violations of the rule–excitations–lead to net vertex charges. In the square ASI studied here there are two ice-rule obeying vertex types as depicted in Fig. 1a,b. The first, known as Type 1 (T_1_) in the standard scheme^22^, has the lowest energy and is the ground state vertex where the islands which are closest together (perpendicular neighbours) have the less energetically-costly interaction of head-to-tail (Fig. 1a). In the field-polarised state, known as Type 2 (T_2_), this more favourable interaction of head-to-tail is between the second nearest neighbours (parallel islands), giving a slightly higher overall energy (Fig. 1b). The difference in energy is due to the reduced dimensionality of the ASI^23^, although the degeneracy can be restored by introducing height offsets so that the system is no longer truly two-dimensional^21,24^, or recently by using slave meso-spins in the middle of the vertex in order to modify the interaction^25^. Starting from an ice-rule obeying state, shown on the left-hand side of Fig. 1a,b, it is possible to flip the macrospin of a nanomagnet in order to change the vertex configurations to Type 3 (T_3_) and consequently violate the “two-in/two-out” ice rule. This results in three-in and one-out state for one vertex (red circle) and three-out one-in for the other (blue circle), as shown on the right-hand side of Fig. 1a,b,which possess opposite net magnetic charges at the vertex and can be considered an emergent monopole-antimonopole pair. These may then move apart by flipping further spins, leaving a flux-carrying chain of vertices that is often termed a Dirac string (see Fig. 2), although it is truly of Nambu form in the two-dimensional system^26^.

The ability to drive these emergent magnetic charges with applied field can be analogous to using electric fields to drive a current of electrical charge carriers. The term ‘magnetricity’ was first coined to describe this effect in the pyrochlore spin ice materials^27,28^. Here we demonstrate the effect of an applied magnetic field on the movement of magnetic charges in the thermally activated regime within our square ASI systems. Many of the imaging methods that have been suited to probe the dynamics of ASI so far exploit the properties of electrons, such as photoemission electron microscopy^19^ and Lorentz transmission electron microscopy^20^. Therefore, there has been a more limited exploration of the thermal fluctuations under a driven magnetic field due to the effect of the field on the electrons in those techniques. Also, due to the high temperatures or low moment materials required for thermal behaviour, magnetic force microscopy (MFM) has not been suitable for dynamic studies of thermally active arrays, since the stray field of the tip would perturb the fluctuating states. The ‘photon-in-photon-out’ nature of the magnetic transmission X-ray microscopy (MTXM) method used here presents a unique opportunity to probe the monopole dynamics as a function of field and temperature. Just as electrons and holes in a semiconductor are driven in opposite directions by an electric field, here we can drive apart the opposite magnetic charges in a pair created from the ice rule state with a magnetic field. By directly imaging this motion using MTXM, we have observed increased mobility of these charges with temperature and coupling strength of the islands, similar to the properties of ionic hopping conduction of electrons in solids^29^. In that case, motion depends on the probability of hopping between sites with a varying potential energy landscape, akin to that which would be expected in a system with a varying degree of disorder in the coercive fields inherent from the patterning process^30^. We find a critical field for the onset of motion of the monopole charges, beyond which the thermally activated drift velocity is linear in drive field. This linear creep regime reveals a reduction in the dimensionality of the system.

## Results

### Magnetic x-ray imaging

Our experiments were performed on square ASI arrays with two different lattice spacings, *a*, of 350 and 400 nm. The 7 nm thick Co_60_Fe_20_B_20_ islands were nominally 250 nm × 80 nm in lateral size and were fabricated on soft x-ray-transparent Si_3_N_4_ membranes. In order to probe the thermally-activated monopole drift motion, we developed an on-membrane heater and thermometer, as shown in Fig. 1c. The lithographically patterned heater can raise the temperature *T* of the thermometer and the enclosed ASI arrays to values in excess of 700 K, repeatedly. The calibration is shown in Fig. 1d and a thermal microscopy image shows an even temperature distribution across the patterned area in Fig. 1e (details in Methods). Our islands are large enough that their macrospins are frozen at room temperature, since the energy barrier for reversal *E*_b _≫ *k*_B_*T*_room_, providing thermally stable states for imaging. Before each image was taken, a current *I*_heater_ was applied for 100 ms to raise the temperature to a value where magnetisation dynamics–under field, if one was applied–can take place. The thermal mass of the membrane is so small that heating and cooling on switching the heater current on and off is effectively instantaneous, freezing the state at the end of the *I*_heater_ pulse ready for imaging. Each image acquired was the average of 8 × 0.8 s exposures.

An example of a raw soft X-ray absorption image taken is shown in Fig. 2a, with the *a = *400 nm ASI array clearly visible. To perform our experiment, we first applied a large saturating field of −73 mT at a temperature of 439 K in order to create a full diagonally polarised T_2_ background, as shown schematically in Fig. 2b. Then a positive injection field of 64 mT (smaller than the room temperature coercivity, *B*_0_ = 74 mT) was applied for 100 ms with zero heater current. This created the injection state, which was engineered so that a low number of monopole-antimonopole excitations were created above the T_2_ background, in order to be able to easily follow their trajectories within the field-of-view. The difference image between the saturated state and an injection is shown in Fig. 2c with its corresponding monopole-antimonopole pair of T_3_ vertices highlighted as red and blue circles in Fig. 2d. They separate via chains of T_1_ vertices connected by reversed islands, which form Nambu strings^26^, shown in the figure as the line of black islands tracing out the path of monopole motion. Once driven with a pulse of magnetic field and temperature, further islands will reverse propagating the charge and lengthening the string as demonstrated in Fig. 2e,f. The variable length of these strings means that they can be considered to be a form of avalanche, with longer strings being less common than shorter ones. Hence, an appropriate analogy with thermally activated drift motion of electrical charges in a variable range hopping regime can be drawn.

**Figure 3:**
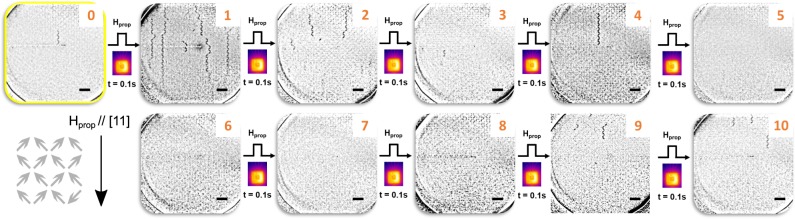
Image sequence for 350 nm lattice spacing sample. The first image (‘0’), marked by a yellow box, is the injection state with a monopole-antimonopole pair joined by a short string. Each subsequent image is the result of a 100 ms heating pulse to a temperature of 488 K under a 62 mT propagation field. Black contrast indicates islands that have reversed with respect to the previous image. Scale bar, bottom right of images, is 1 *μ*m.

A full sequence of difference images is presented in Fig. 3 for the *a* = 350 nm array. Highlighted in the yellow box of the figure is the injection state, which contains one chain of reversed islands with a length of 6 islands. Each chain marks the separation path of a monopole-antimonopole pair at its ends. Just before each subsequent image a propagation field *H*_prop_ = 62 mT and a temperature of 488 K was applied for 100 ms, after which the array was frozen down to room temperature and the resultant string propagation was imaged. This field value is too small to cause any change in the state of the array at room temperature, it is necessary to heat the array in order to induce thermally-activated dynamics under field. It can be seen from the image sequence that there are many initial events after the first propagation field pulse where many strings can be seen within the field of view which we attribute to the finite size of the array and the field of view. After this there are fewer per image but they tend to have string lengths, *L* > 4 islands. This was found to be in contrast to an image sequence with the same propagation field but a lower temperature of 467 K, in that sequence there were many fewer reversals during the same measurement time (see Supplementary S1 for an image sequence at *T* = 467 K).

### Monopole velocity measurement

**Figure 4:**
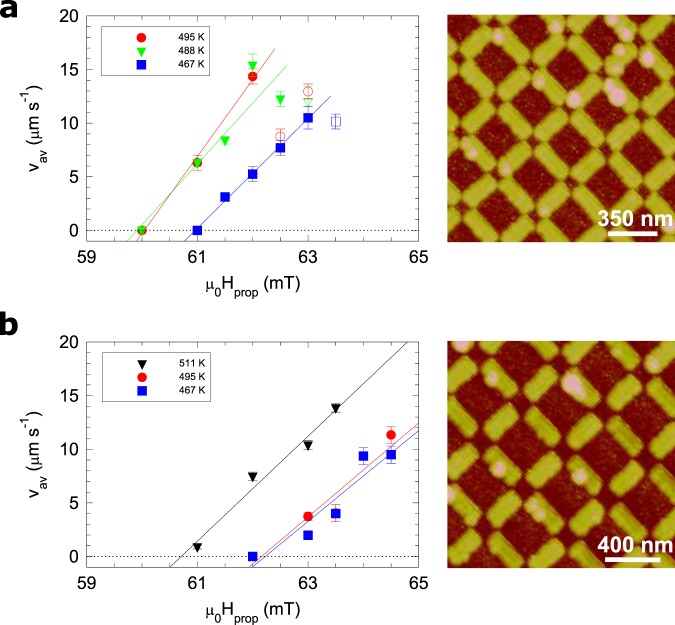
Monopole velocities. (**a**) The measured velocity of the monopole excitations in the *a* = 350 nm array is plotted as a function of the propagation field. Higher velocities are reached at lower fields for increased temperature. Data points at higher fields, in the regime when strings start to overlap (indicated by open symbols), are neglected in the fits. (**b**) For the more weakly interacting, *a* = 400 nm, sample where the velocities are plotted on the same scale for comparison but at a slightly extended temperature range. The solid lines show straight line fits to the data in the linear regime just above the critical field for the onset of motion. An atomic force micrograph for each sample is also shown on the right of each panel.

The average monopole velocity *v*_av_ was measured in each case, which was defined as the average string length per frame over the entire pulse sequence (i.e. a total of 10 pulses for a total duration of 1 s, during which thermally activated dynamics took place). As pointed out previously, for the 350 nm sample the initial image (frame 1), in general, had more strings and a discussion of a different averaging method without this frame is included in the Supplementary S4. Using frames 1 to 10 for the averaging, we observed an average velocity of 44 lattice hops s^−1^ at *T* = 488 K, compared to 18.5 lattice hops s^−1^ for *T* = 467 K. The mean observed string length was also longer for the higher temperature, 8.1 ± 0.6 islands compared to 5.0 ± 0.5 islands. As previously mentioned, the separation of these oppositely charged emergent monopoles can be likened to the flow of electric charge, and in order to draw a parallel to this physics we measured the average velocity using various propagation fields at different temperatures. The results are shown in Fig. 4a for the *a* = 350 nm array. The velocity for each temperature has a similar form: there is a critical field below which no monopole motion was observed, after which there is a regime that is a few mT wide where the velocity is linear in field. At higher fields an apparent departure from linearity occurs, due to strings growing so rapidly that they start to overlap, which makes the measurement of individual monopole velocities unreliable (an example of a sequence exhibiting this type of behaviour can be found in Supplementary S2).

We performed an equivalent set of measurements for the less strongly interacting ASI, in which the islands were the same size but the lattice spacing was increased to 400 nm. The most striking difference is the average string length is greatly reduced to 2.0 ± 0.1 islands, less than a quarter of that observed for the 350 nm lattice spacing for the same temperature and propagation field (*T* = 488 K and *H*_prop_ 62 mT). The average emergent monopole velocity is more than ten times slower than the 350 nm lattice: 2.0 lattice hops s^−1^ c.f. 44 lattice hops s^−1^. At the lower temperature, *T* = 467 K we observed no monopole motion at all in the 400 nm lattice. The same analysis to obtain the average velocity at different temperatures was employed and the results are plotted in Fig. 4b. For the larger lattice spacing the propagation dynamics are shifted to a higher field range. Also, the velocities are in general much slower than those for the more closely coupled array. However, at the highest temperature, 511 K, similar velocities can be reached to those of the lowest temperature in the 350 nm sample. A comparative image sequence for the two lattice spacings is shown in Supplementary S2 and S3.

The principal feature of the datasets in Fig. 4 is the regime in which velocity is linear in driving field after the onset of monopole motion at a critical field *H*_crit_. This can be described as

1 $${v}_{{\rm{av}}}={\mu }_{{\rm{m}}}({H}_{{\rm{prop}}}-{H}_{{\rm{crit}}}),$$

in which *μ*_m_ is a monopole mobility. We have defined this in analogy with electrical charge carrier mobility *μ* in an electric field *E* in semiconductor physics, where *μ* = *dv*/*dℰ*. The propagation field, *H*_prop_, is analogous to the electric field, providing the “tilted” energy landscape to drive charge drift motion. This tilt defines the preferred direction of propagation, which is the reason why the strings expand vertically in the image. Both *μ*_m_ and *H*_crit_ were determined for each dataset in Fig. 4 by fitting a straight line to the data in the linear regime. For the *a* = 350 nm sample this was only done for the low field region of the data due to too many reversals within the field of view to extract a meaningful average velocity, as previously mentioned.

**Figure 5:**
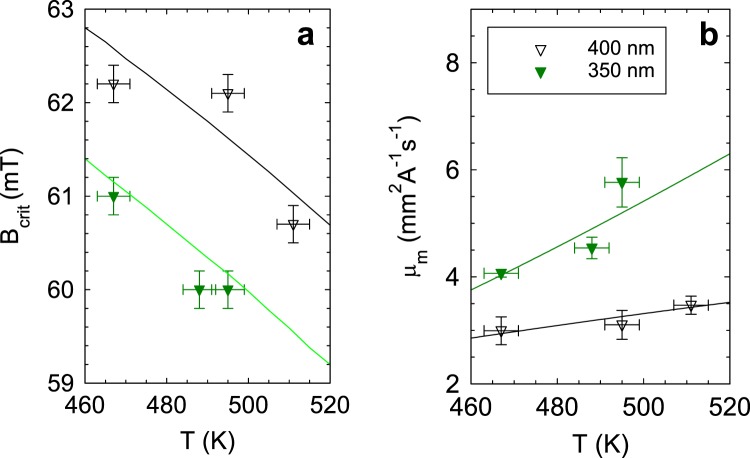
Critical field and mobility for monopole drift motion. (**a**), The data points show the critical field, *B*_crit_ = *μ*_0_*H*_crit_, for the onset of monopole motion for arrays of two different strengths of interaction, extracted from the fits in Fig. 4. The solid lines are the values from the modified Bean-Livingston theory. (**b**) The data points show the magnetic mobility *μ*_m_ extracted from the fits in Fig. 4, also plotted for each array. The solid lines are fits of Eq. 5 to the data. In both cases the solid symbols are for the *a* = 350 nm and the open symbols are for the *a* = 400 nm array.

The results of these fits are shown in Fig. 5, in which we display the temperature dependence of both *B*_crit_ = *μ*_0_*H*_crit_ (Fig. 5a) and *μ*_m_ (Fig. 5b) for each of the two values of *a*. We see that *B*_crit_ reduces as the temperature rises and is less for a more strongly coupled array. On the other hand, *μ*_m_, the monopole mobility, rises as the array is warmed, and is larger for a more strongly coupled array.

### Critical field for onset of monopole motion

In order to describe the onset of monopole motion at *H*_crit_, we have considered that the thermally activated reversal rate of the nanoislands is limited by the rate of hopping over the shape anisotropy energy barrier^31^. For zero applied field, this barrier is given by *E*_0_ = *KV*, where $K=\frac{1}{2}{\mu }_{0}{N}_{D}{M}_{{\rm{S}}}^{2}$, with *N*_*D*_ ≈ 0.1 being the difference between the demagnetisation factors for the easy and hard axes^32^, and *M*_S_ = 1.0 ± 0.1 MA/m is the measured saturation magnetisation of the Co_60_Fe_20_B_20_. The barrier for each nanoisland must be overcome by thermal fluctuations in order to propagate the monopole excitations. The key point is that the barrier is reduced by a magnetic field^33^, which in this case is a combination of the applied field *H*_prop_ and local coupling fields from neighbouring islands.

We can describe the velocity of the emergent monopoles by considering the rate of expansion of a string in both directions. In that case we have a rate *ṅ*_*t*_ for the string to expand to the top and the same for the bottom *ṅ*_*b*_. We can then describe the overall rate of growth of the string, $\dot{L}$, as:

2 $$\dot{L}={\dot{n}}_{t}+{\dot{n}}_{b},$$

where ${\dot{n}}_{t,b}={f}_{0}({e}^{-{E}_{ > }\beta }+{e}^{-{E}_{ < }\beta })$, *f*_0_ is an attempt frequency, and *β* = 1/*k*_B_*T*. The two different energy terms here describe in one case the lengthening of the string, *E*_>_, but also include the finite probability of a reversal against the field and a subsequent contraction of the string, *E*_<_. We can define these using the Bean-Livingston field-adjusted barrier height^33^:

3 $${E}_{ > }={E}_{0}{(1-\frac{[\frac{\Delta }{m}+{B}_{{\rm{prop}}}]}{{B}_{0}})}^{n}$$

4 $${E}_{ < }={E}_{0}{(1+\frac{[\frac{\Delta }{m}+{B}_{{\rm{prop}}}]}{{B}_{0}})}^{n}$$

where *E*_0_ is the zero field energy barrier of the system described above, Δ is the energy difference between the initial vertex type and the final vertex type *E*(T_*i*_) − *E*(T_*f*_), *m* = *M*_*S*_*V* is the magnetic moment of the island, *B*_prop_ = *μ*_0_*H*_prop_, *B*_0_ is the measured room temperature coercivity, and *n* = 1.5 from the geometry of the field with respect to the easy axis of the islands^34^. Here the terms involving Δ are additions to the original model^33^ that we have used to represent the interactions between the islands.

Using Eqs 2–4, we were able to simulate the velocity data as a function of the propagation field. From this we could use the lowest limit of one flip in the entire measurement time as the value of field for the onset of motion, *H*_crit_, below which we would not observe any island flips beyond the injection state (*i.e. v*(*H*_crit_) = 1 lattice hop per second = 0.35 or 0.40 *μ*ms^−1^) and reproduce the temperature dependence of the measured critical fields as shown by the lines plotted in Fig. 5a. It should be noted in order to get good agreement with the experimental data, the temperature-corrected *M*_S_ value from Bloch’s law^35^ *M*(*T*) = *M*(0)[1 − (*T*/*T*_C_)^3/2^], with *T*_C_ = 1200 K, was used for the Co_60_Fe_20_B_20_. In the majority of cases, the strings travel in the direction of the applied field which means Δ = *E*(T_2_)−*E*(T_1_), this was estimated from numerical micromagnetic simulations, carried out using the OOMMF code^36^, as 3.3 × 10^−19^ J and 1.87 × 10^−19^ J for *a* = 350 nm and 400 nm, respectively (see Methods section). We also found the hopping rate associated with *E*_<_ (jumps against the field direction) to be small enough to have a negligible effect on the results. This model correctly reproduces both the temperature and coupling strength dependence of *Hcrit*. Unfortunately, this model predicts an exponential dependence of velocity on drive field beyond *Hcrit*, much sharper than the linear behaviour observed in the experiment (see Supplementary S5). The mobility thus needs to be described using an alternative approach, which will be outlined in the following section.

### Monopole mobility

In Eq. 1 we define the monopole mobility as *μ*_m_ = *dv*_av_/*dH*_prop_. An equivalent quantity has been used to describe field-driven domain wall motion. For instance, Beach *et al*. have defined a magnetic mobility in the study of domain wall velocity as a function of applied field in Permalloy nanowires^37^. In their case they found the velocity-field curve was characterised by two regions of linearity; the low field region which has a significantly larger mobility than the high field region, in between this a negative differential mobility is observed and attributed to the Walker breakdown. That linear behaviour was in the viscous flow or precessional regimes in which thermal activation plays no role. Rather, the appropriate analogy for our thermally activated results is the so-called creep regime in which domain wall motion is frozen at 0 K but can be thermally activated at finite temperature^38^. This creep motion is usually described by a model in which a one-dimensional elastic domain wall moves through a disordered two-dimensional energy landscape that predicts a non-linear creep law in which *v*_av_ ∼ exp(*H*^−1/4^)), which is experimentally well-obeyed^39^.

Nevertheless, a crossover to linear behaviour for domain wall creep velocity has been identified recently for walls in sufficiently narrow nanowires^40^. This has been theoretically explained as being due to the reduction in dimension: the domain wall becomes a zero-dimensional object moving in a one-dimensional energy landscape^41^. That work predicts a mobility for field-driven motion that has the form

5 $${\mu }_{{\rm{m}}}=A\exp (\frac{-{\varepsilon }^{2}}{{k}_{{\rm{B}}}^{2}{T}^{2}})$$

where the prefactor *A* = 2*μ*_0_*M*_S_/Γ in which Γ is a measure of magnetic “friction”, *ε* is the standard deviation of the random one-dimensional energy landscape, and *k*_B_ is the Boltzmann constant. Since our monopoles, just like domain walls, are localised magnetic excitations over a ground state, we fitted this expression to our mobility data for both values of *a*. The results are shown in Fig. 5b.

The prefactor *A* is hard to determine accurately: the results of the fitting yield *A* = 41 ± 42 mm^2^ A^−1^ s^−1^ and 7 ± 3 mm^2^ A^−1^ s^−1^ for *a* = 350 nm and 400 nm, respectively. Any difference here is not meaningful since we are trying to fit with a function which saturates to the value *A* at large *T* (i.e. *T* ≫ *ε*), and the data are quite far from that limit.

The results for *ε* are more meaningful. Our fits return values of *ε* = (9 ± 2)× 10^−21^ J and (6 ± 1)× 10^−21^ J for *a* = 350 nm and 400 nm, respectively. These energies correspond to temperatures *ε*/*k*_B_ of 600 ± 200 K and 400 ± 100 K, respectively. Stronger coupling appears to help smooth out the variations in the energy landscape, leading to higher mobility. These temperatures are comparable to the ones needed to obtain thermally activated creep in our experiments. This shows the need for thermal fluctuations on the scale of the spatial fluctuations in the one-dimensional energy landscapes to obtain linear creep motion of monopoles on laboratory timescales.

It is physically reasonable that the mobility rises sharply as the temperature approaches the scale of the typical spatial fluctuation in the energy landscape, so the temperature dependence of *μ*_m_ can be understood on this basis. The fact that the mobility is higher for more strongly coupled samples can be attributed to the avalanche nature of the motion. The flipping of one island is more likely to cause the next island in the chain to flip if the coupling is stronger, leading to the monopole propagating further during that avalanche event and leading to a higher drift velocity and hence higher mobility.

The fact that a linear dependence of *v*_av_(*H*_prop_) is observed thus means that the monopoles in the square ASI can be treated as zero dimensional point-like objects and experience a reduction in the effective dimension of their environment from two dimensions to one. A similar dimensional reduction was previously inferred for the kagomé ice on the basis of the avalanche statistics departing from a Gutenberg-Richter-type power law^19,42^.

### Zig-zag and side-jump statistics

**Figure 6:**
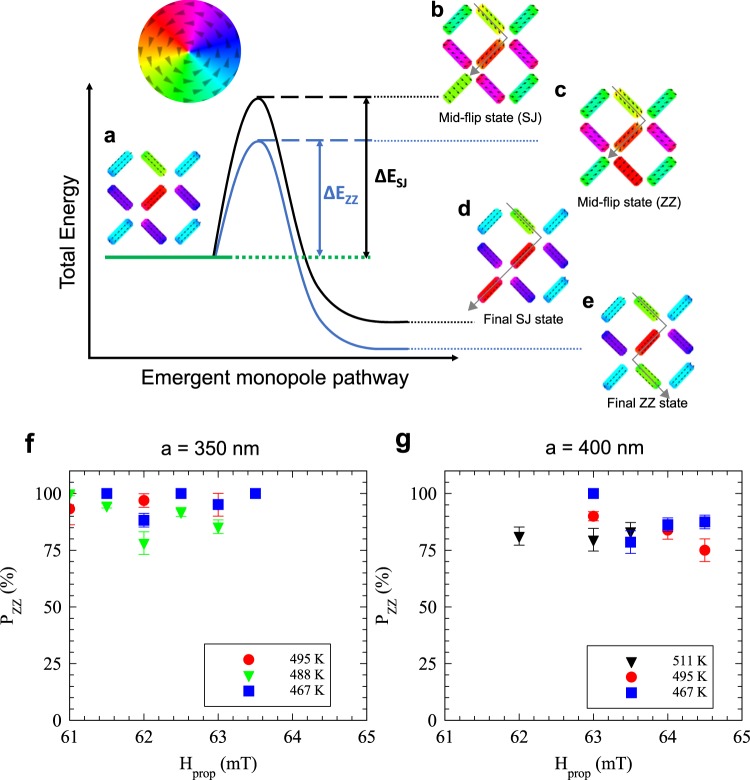
Energy level diagram and probability of monopole pathways. (**a**) The initial state of a string with a relaxed magnetic state before propagation of the monopole excitation. (**b**) The simulated mid-flip state during a side-jump propagation, the island in the middle of flipping has its magnetisation pointing along its hard axis, which is green. (**c**) The simulated mid-flip state during a zig-zag propagation, the island in the middle of flipping has its magnetisation pointing along its hard axis, which is red. (**d**) Shows the final SJ state where the string has been propagated to the parallel, next nearest neighbour, island. (**e**) Shows the final ZZ state where the string has been propagated to the perpendicular, nearest neighbour, island. The SJ and ZZ pathways have been outlined with grey arrows. (**f,g**) The probability of observing a ZZ event (*P*_*ZZ*_) for all image sequences in the 350 nm and 400 nm array, respectively.

Finally, we consider the difference in the probability that the magnetic charges would propagate to the next-nearest-neighbour which we will refer to as a side-jump (SJ) event (Fig. 6d) compared with the more frequently observed nearest-neighbor propagation which we will call a zig-zag (ZZ) event (Fig. 6e). These two possible energetic pathways are illustrated in Fig. 6. Starting from an initial state shown in Fig. 6a, the system requires thermal activation in order to flip the next island and propagate the string excitation further into the T_2_ background, either Δ*E*_ZZ_ or Δ*E*_SJ_. We can calculate this barrier using OOMMF by comparing the total energy of the initial state with the “mid-flip” states shown in Fig. 6b,c. In those states, the nearest neighbour’s (ZZ) or the next nearest neighbour’s (SJ) magnetisation was fixed along the element’s hard axis, as shown in the figure. As it is thermally activated we can use Boltzmann factors to determine the relative probabilities. The continued zig-zag event ought to be more likely than the side-jump event by a factor of exp(−*E*_ZZ_/*k*T)/exp(−*E*_SJ_/*k*T) = exp([*E*_*SJ*_ − *E*_*ZZ*_]/*k*T)). The data at different fields and temperatures for each lattice are shown in Fig. 6f,g, we took the statistical average for each lattice as there is little effect on the probabilities in the temperature and applied field range measured, as judged by the scatter in the data. From experiment, we found the likelihood of zig-zag propagation in the *a* = 350 nm lattice, *P*_ZZ_(350*nm*) = 94 ± 2% which gave a ratio *P*_ZZ_/*P*_SJ_(350 nm) = 17 ± 6. In the *a* = 400 nm lattice, we found it was slightly more likely to observe a side-jump propagation which reduced the probability of zig-zag propagation in that lattice; *P*_ZZ_(400 *nm*) = 84 ± 2% which gave a reduced ratio *P*_ZZ_/*P*_SJ_(400 nm) = 5 ± 1. In order to get good agreement with the simulated energy barrier ratios using OOMMF, *P*_ZZ_/*P*_SJ_(350 nm) = 16.9 and *P*_ZZ_/*P*_SJ_(400 nm) = 4.1, we had to increase the theoretical temperature compared to the experiment by a factor ≈ 10. This discrepancy is likely due to activation volumes in the real experiment being smaller than the full island volumes used in the simulation. This is an effect we have observed previously in other ASI systems^8,31^. The simulation still offers a method for predicting the relative probabilities of the likelihood of a certain type of motion in these nanopatterned ASI systems and could be used as a tool for designing functional networks of mobile magnetic charges.

## Conclusion

We have been able to image directly the thermally-activated drift motion of magnetic monopoles in artificial spin ices using a newly developed on-membrane heating device and magnetic transmission X-ray microscopy. The motion only occurs above a critical field and takes the form of one-dimensional strings that can be interpreted as Nambu strings. We have measured the drift velocity of the magnetic monopole charges as a function of drive magnetic field and temperature for arrays of different coupling strength from these images. This revealed the temperature and coupling strength dependence of both the critical field for the onset of motion and also their mobility, defined by analogy with the mobility of electric charges under an electric drive field.

The temperature and coupling strength dependence of the critical field exhibit exponential behaviour that can be described by a Bean-Livingston model modified to include coupling fields between the elements. This model is one that is commonly used to describe the thermally activated reversal of a nanomagnet.

The monopole drift motion is also due to thermal activation, but is found to be linear, rather than exponential, in drive magnetic field. Drawing inspiration from the crossover of domain wall creep motion from exponential to linear behaviour as the dimensionality is reduced, the equivalent dependences of the mobility have been described using a model originally developed to describe this linear form of creep motion. This allows us to extract the scale of the variations in the energy landscape through which the monopole charges propagate, which are smoothed out by stronger inter-island coupling.

These results show that the flow of currents of ‘magnetricity’ can be directly imaged at the individual charge carrier level in artificial spin ices and that dimensional reduction for avalanches is a property of the square ice as well as the kagomé ice.

## Methods

### Growth and characterisation

The Co_60_Fe_20_B_20_ alloy was first sputtered onto 100 nm-thick Si_3_N_4_ membranes. Magnetisation of the thin film was measured using a vibrating sample magnetometer. After deposition, the membranes were spin coated with ZEP520A: anisole (1:1) to a resist film thickness ≈ 140 nm. The ASI arrays were then defined using electron beam lithography. They were written using an electron dose of 343 *μ*C/cm^2^. The pattern was developed for 70 s in N50 solution. Ti was evaporated into the pattern and lifted off to provide a hard mask for broad-beam Ar ion milling. The sample was milled for 80 s to remove the unwanted CoFeB film around the mask and leave only the CoFeB in the desired spin ice array pattern. A PMMA-based bilayer resist and another electron beam lithography step were used to create the on-membrane heater and thermometer pattern, after which Pt was evaporated at a thickness of 60 nm to create the heater and thermometer. The calibration of the thermometer was done by submerging in a non-conductive liquid, the liquid was heated whilst the resistance of the thermometer and temperature of the liquid were measured (further details can be found in ref.^43^). Thermal imaging was carried out using a FLIR thermal imaging camera with a macro lens to check the temperature distribution across the membrane.

### Soft X-ray magnetic microscopy

All the MTXM experiments were carried out at the full-field soft X-ray microscope, XM-1, located at beamline 6.1.2 at the Advanced Light Source. This microscope has a spatial resolution of about 15 nm, images can be recorded with an exposure time of a few seconds and it covers a several micrometer field of view^44^. All images were taken at the Co L_3_ edge (778 eV) with circularly polarised X-rays of a fixed helicity. This provided strong X-ray magnetic circular dichroism (XMCD) contrast arising from the high Co content of the Co_60_Fe_20_B_20_ alloy. A nanomagnet whose moment is oriented parallel to the X-ray propagation vector will have absorption different from one that has its anti-parallel, which provides the magnetic contrast mechanism. Since our islands are magnetised in-plane, the sample was tilted at an angle of 30° from normal incidence to give a magnetisation component along the beam direction. A back-thinned, back-illuminated 2048 × 2048 pixel CCD camera acts as a detector to form the image, so the absorption is directly measured. A sample raw CCD image is shown in Fig. 2a. A contrast image is obtained by dividing two consecutive absorption images, darker contrast indicates those islands that have switched their moment orientation, as shown in Fig. 2b. The sample is aligned in such a way that the field is applied in the film plane and along a diagonal of the ASI array, so that all islands have their magnetic easy axis, which is defined by their elongated shape, at 45° to the propagation field direction, *H*_prop_.

### Simulations

Simulations were carried out using finite element micromagnetic calculations by means of the 3-dimensional Oxsii option of the OOMMF code, in order to calculate the exchange and demagnetizing energies of the different vertex configurations. Rectangular nano-island shapes with rounded edges were used, discretized into 2 × 2 × 3.5 nm^3^ unit cells. The saturation magnetization *M*_S_ was determined to be 1.0 +  0.1 MA/m experimentally; the exchange stiffness constant *A* used was 27.5 pJ/m, estimated from a stoichiometric average of that of Co (30 pJ/m) and Fe (20 pJ/m); and the magnetocrystalline anisotropy constant *K* was assumed to be zero in this amorphous soft magnet. The Gilbert damping coefficient *α* was set to the unphysically high value of 0.5, allowing for rapid convergence (convergence criterion used: *dm*/*dt <* 0.1 deg/ns), after obtaining similar vertex energies using 0.5 and 0.016 (where the latter simulation time is much longer). For the zig-zag and side-jump statistics sections, the energy differences for the side-jump and zig-zag events have been calculated by relaxing the magnetization states shown in Fig. 6a (initial state) and Fig. 6b,c (mid-flip states). For the latter case, the reversing nano-island has its magnetic moments fixed along the hard axis, as this is the maximum energy barrier that the system has to overcome when going from the initial to the final state in each case. The fixing of the magnetic moments to the hard axis has been done using the OOMMF built-in function“fixed_spins” for that particular element.

## Supplementary information

Supplementary material

## Data Availability

The data sets generated and/or analysed during this study are publicly available in the University of Leeds Research Data Repository, 10.5518/585.
