# Author Correction: Thermally and field-driven mobility of emergent magnetic charges in square artificial spin ice

**DOI:** 10.1038/s41598-020-60497-2

**Published:** 2020-02-19

**Authors:** Sophie A. Morley, Jose Maria Porro, Aleš Hrabec, Mark C. Rosamond, Diego Alba Venero, Edmund H. Linfield, Gavin Burnell, Mi-Young Im, Peter Fischer, Sean Langridge, Christopher H. Marrows

**Affiliations:** 1 School of Physics and Astronomy, University of Leeds, Leeds, LS2 9JT United Kingdom; 2 Physics Department, University of California Santa Cruz, Santa Cruz, CA 95064 USA; 3 Advanced Light Source, Lawrence Berkeley National Laboratory, 1 Cyclotron Road, Berkeley, CA 94720 USA; 4 ISIS Neutron and Muon Source, STFC Rutherford Appleton Laboratory, Chilton, Didcot, Oxon OX11 0QX United Kingdom; 5 BCMaterials, Basque Center for Materials, Applications and Nanostructures, 48940 Leioa, Spain; 6 Ikerbasque, Basque Foundation for Science, 48013 Bilbao, Spain; 7 School of Electronic and Electrical Engineering, University of Leeds, Leeds, LS2 9JT United Kingdom; 8 Center for X-ray Optics, Lawrence Berkeley National Laboratory, 1 Cyclotron Road, Berkeley, CA 94720 USA; 9 Daegu Gyeongbuk Institute of Science and Technology, Daegu, 711-873 Korea; 10 Materials Sciences Division, Lawrence Berkeley National Laboratory, 1 Cyclotron Road, Berkeley, CA 94720 USA

Correction to: *Scientific Reports* 10.1038/s41598-019-52460-7, published online 05 November 2019

The original version of this Article contained an error in Affiliation 6, which was incorrectly given as ‘Ikerbasque, Basque Foundation for Science, 48013 Bilbao, SpainIkerbasque, Basque Foundation for Science, 48013, Bilbao, Spain’. The correct affiliation is listed below:

Ikerbasque, Basque Foundation for Science, 48013, Bilbao, Spain

In addition, Edmund H. Linfield was incorrectly affiliated with ‘Ikerbasque, Basque Foundation for Science, 48013 Bilbao, SpainIkerbasque, Basque Foundation for Science, 48013, Bilbao, Spain’. The correct affiliation is listed below.

School of Electronic and Electrical Engineering, University of Leeds, Leeds, LS2 9JT, United Kingdom

These errors have now been corrected in the PDF and HTML versions of the Article.

