# Supplementary material for: Thermally and field-driven mobility of emergent magnetic charges in square artificial spin ice
Source: Sci Rep. 2019 Nov 5;9:15989. doi: 10.1038/s41598-019-52460-7 (PMC6831649; doi:10.1038/s41598-019-52460-7)
Supplement: Supplementary file 1 — Supplementary material [file 41598_2019_52460_MOESM1_ESM.docx]

**Thermally and field-driven mobility of emergent magnetic charges in square artificial spin ice (supplementary material)**

Sophie A. Morley, Jose Maria Porro, Ales Hrabec, Mark C. Rosamond, Diego Alba Venero, Edmund H. Linfield, Gavin Burnell, Mi-Young Im, Peter J. Fischer, Sean Langridge, and Christopher H. Marrows

An example of an image sequence for the a = 350 nm sample is shown for T = 467 K in Fig S1 and T = 495 K in Fig. S2, compared to the T = 488 K data shown in Fig. 3 of the manuscript. A similar overall behavior is observed but a with lower average velocity in S1 and higher in S2. Due to the large number of reversals, more than half the total islands, in image sequence S2, this is an example of data where an unreliable measure of velocity was obtained. For this reason, the velocity extracted from a sequence such as this is plotted in the open symbols in Fig. 4 of the manuscript and was not used in the linear fits used to determine mobility of the emergent magnetic charges. Also shown for comparison in Fig S3, is the image sequence for the less interacting sample, a = 400 nm, for the same measurement parameters as those for the smaller lattice spacing shown in S2.

As can be seen from the image sequences, the initial frame (“Frame #1”) tends to have more reversed islands compared with subsequent frames. This means the average velocity of this frame is higher than subsequent frames. Therefore, we compared different velocity averaging methods to see if this influenced the behavior of the velocity as a function of propagation field and the subsequent extracted mobilities and their temperature dependence. A comparison of the data averaged with and without the first frame is shown in Fig. S4a for the 350 nm lattice. We observe the same linear behavior of the velocity with applied propagation field. It can be seen most clearly from the T = 467 K data that the velocity still follows a clear linear trend with the propagation field, but with lower average values. The extracted mobilities from the fitted dashed lines (Fig S4a) which don’t include frame 1 are plotted in Fig S4b along with those which do. Again, the qualitative trend is the same but with a different scaling factor. The mobility as a function of temperature is also still fit with the linear creep model, as shown. We do extract a very different value for the fitted prefactor A. However, we do not draw any conclusions from A in the manuscript as we concede to the limitations of our data and the fitting in this respect. The more meaningful value that is extracted and discussed, ε, is an order of magnitude different. Which meant the temperatures discussed in the main text were higher for the alternative averaging method, i.e. 850 K cf. 640 K. The averaging method has no effect on the B_crit_ fields as they are defined as when no monopole motion is observed in the experiment, so it is unaffected by the averaging method.


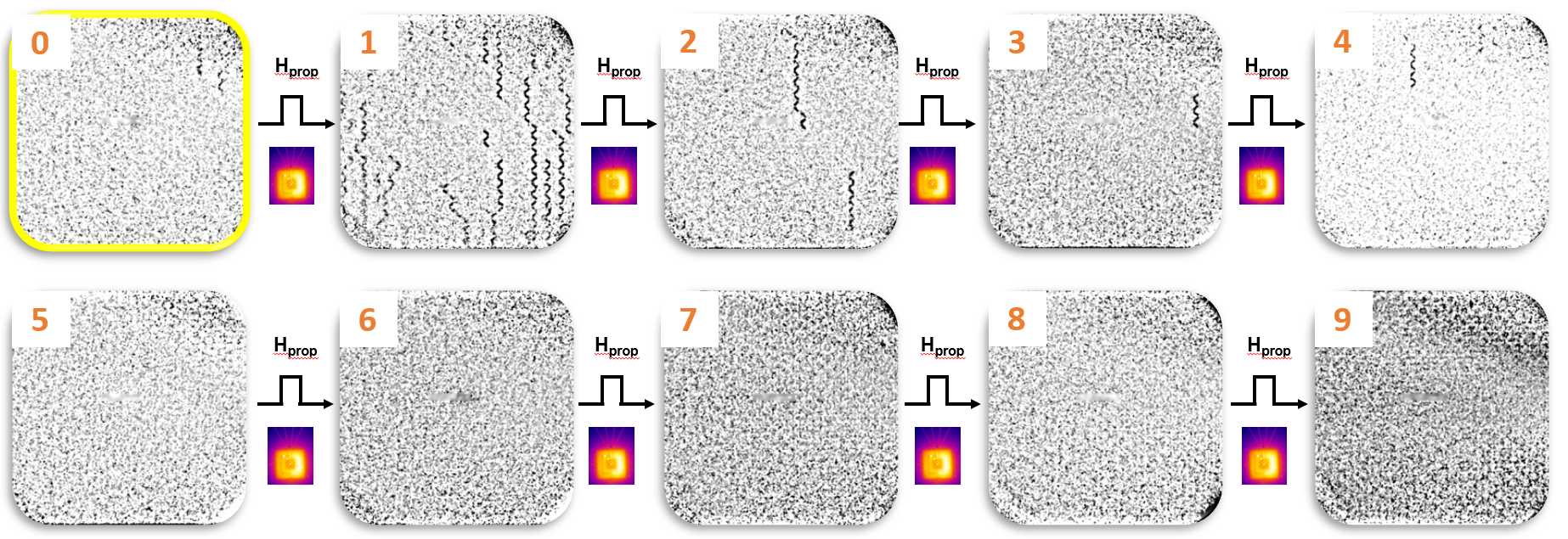


**Figure S1.** Image sequence for 350 nm lattice spacing sample at T = 467 K, H_prop_ = 63 mT. The first image (‘0’), marked by a yellow box, is the injection state with a two monopole-antimonopole pairs joined by short strings. Each subsequent image is the result of a 100 ms heating pulse to a temperature of 467 K under a 63 mT propagation field. Black contrast indicates islands that have reversed with respect to the previous image.


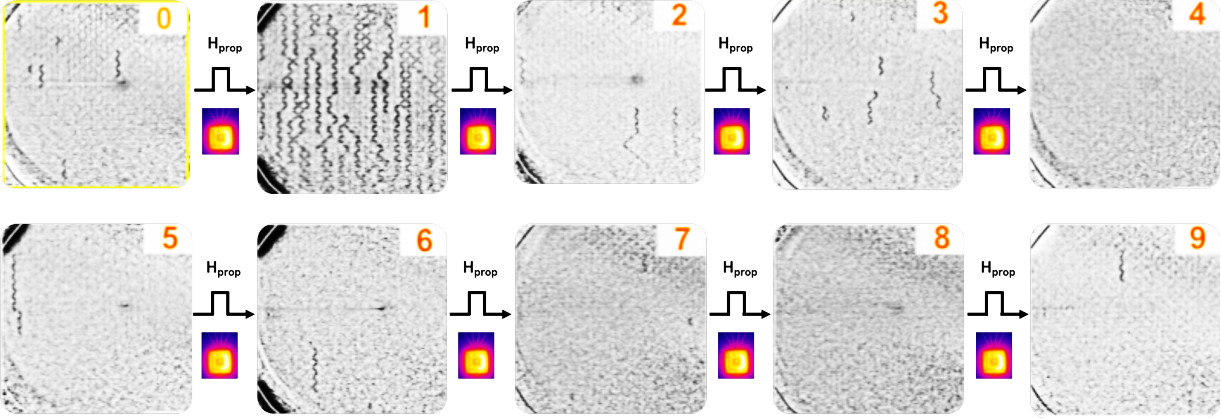


**Figure S2.** Image sequence for 350 nm lattice spacing sample at T = 495 K, H_prop_ = 63 mT. The first image (‘0’), marked by a yellow box, is the injection state. Each subsequent image is the result of a 100 ms heating pulse to a temperature of 495 K under a 63 mT propagation field.


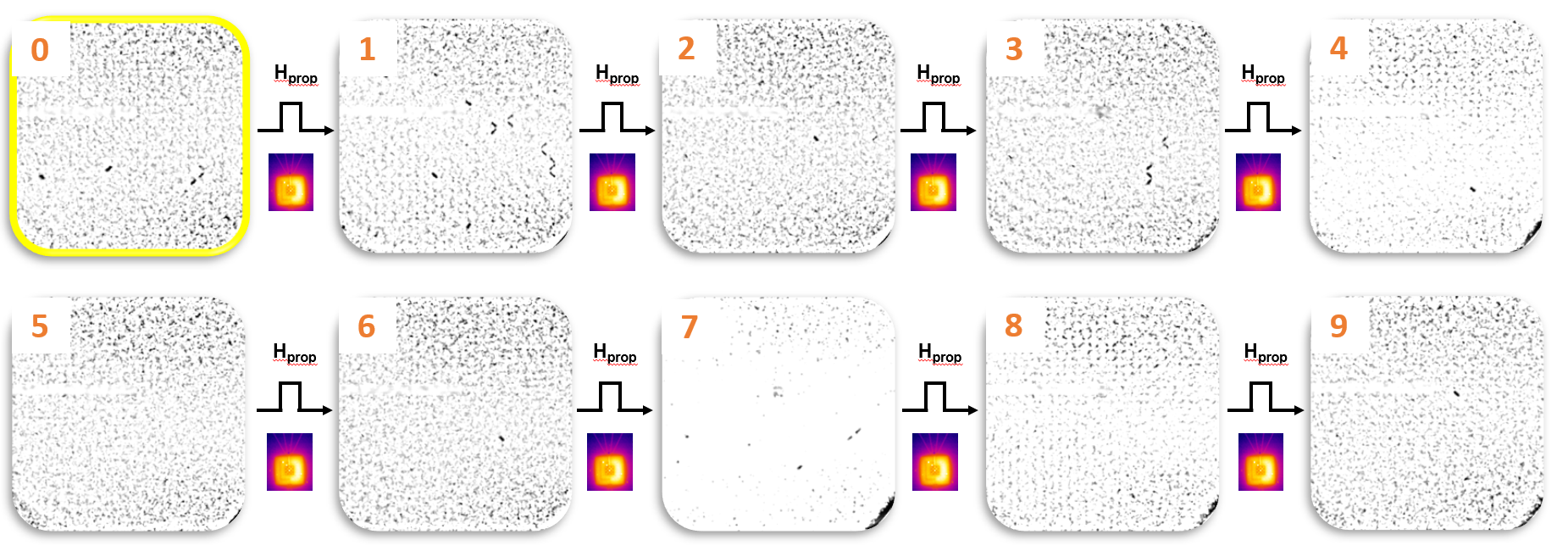


**Figure S3.** Image sequence for 400 nm lattice spacing sample at T = 495 K, H_prop_ = 63 mT. The first image (‘0’), marked by a yellow box, is the injection state. Each subsequent image is the result of a 100 ms heating pulse to a temperature of 495 K under a 63 mT propagation field.


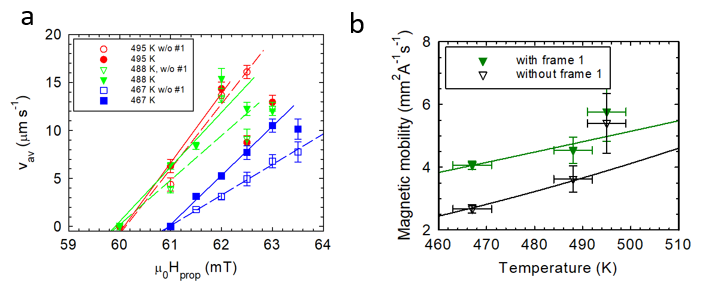


**Figure S4.** Different averaging methods for velocity. a, The velocity data calculated for the two averaging methods with frame 1 (solid symbols) and without frame 1 (open symbols). Fits to the data from averaging which includes frame 1 are solid lines and fits with frame 1 excluded are the dashed lines. **b,** The extracted mobilities from the different averaging methods showing a similar temperature dependence but with different scaling, fits are to Eq. 5 in the manuscript.


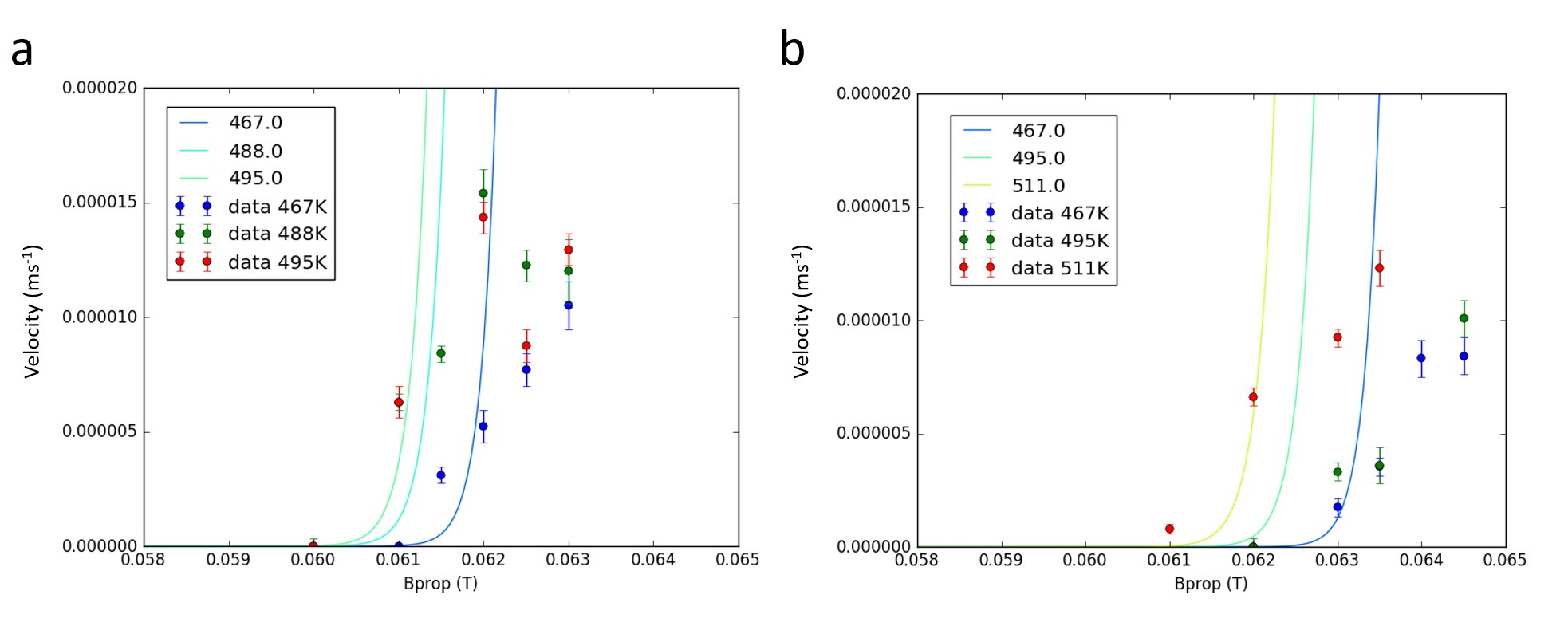


**Figure S5.** Adjusted Bean-Livingston model for B_crit_ prediction. The data for velocity as a function of propagation field, B_prop_, for **a**, the 350 nm lattice and **b**, the 400 nm lattice plotted as points. The velocity as predicted by the adjusted Bean-Livingston model is plotted as lines.

In Fig. S5 we show the measured average velocities, as shown in Fig. 4 of the manuscript, along with the theoretically predicted velocity using the adjusted Bean-Livingston model:

${v(B)=\frac{1}{2}f_{0}(e}^{-E_{inc} \beta}-e^{-E_{dec}\beta})$,

where f_0_ is the attempt frequency, β = 1/kT, E_inc_ and E_dec_ are defined in equations (3) and (4) of the manuscript, respectively. The intercept with the minimum velocity we could measure in the two lattices over the total 1s measurement time i.e., v(B_crit_) = 1 lattice hop per second = 0.35 or 0.40 µ ms^−1^, was used to numerically determine the B_crit_ values at different temperatures, which is the theory plotted by the line in Fig. 5a of the manuscript. Also seen in Fig. S5, the model can predict the critical field values for the onset of motion well, but it predicts an exponential increase of velocity as a function of propagation field. A linear relationship is much more appropriate to describe the data which leads to the linear creep model used in the study. All values used to plot the lines in Fig. S5 are listed in table S1.

| **Model parameter** | **Value** |
| --- | --- |
| *M_0_* (MA/m) | 1.0 |
| *T_C_* (K) | 1200 |
| *N_D_* | 0.1 |
| *n* | 1.5 |
| *B_0_* (T) | 0.0741 |
| *V* (nm^3^) | 80 × 250 × 7 |
| *f_0_* (Hz) | 1e9 |
| *a* (nm) | 350,  400 |
| *Δ* (× 10 ^-19^ J) | 3.30 (a = 350 nm),  1.87 (a = 400 nm) |

**Table S1.** All values for the model parameters used were exactly the same for both lattices except for *a* and *Δ.* These values were either measured experimentally, taken from theory or simulated using OOMMF, as detailed in the main text and Methods section.
